# Development and Characterisation of a Topical Methyl Salicylate Patch: Effect of Solvents on Adhesion and Skin Permeation

**DOI:** 10.3390/pharmaceutics14112491

**Published:** 2022-11-17

**Authors:** Soo Chin Yeoh, Poh Lee Loh, Vikneswaran Murugaiyah, Choon Fu Goh

**Affiliations:** 1 Discipline of Pharmaceutical Technology, School of Pharmaceutical Sciences, Universiti Sains Malaysia, Minden 11800, Penang, Malaysia; 2 THP Medical Sdn Bhd, 1209, Jalan Perindustrian Bukit Minyak 18, Kawasan Perindustrian Bukit Minyak, Simpang Ampat 14100, Penang, Malaysia; 3 Discipline of Pharmacology, School of Pharmaceutical Sciences, Universiti Sains Malaysia, Minden 11800, Penang, Malaysia; 4 Centre for Drug Research, Universiti Sains Malaysia, Minden 11800, Penang, Malaysia

**Keywords:** methyl salicylate, topical drug delivery, solvents, patch, adhesion, skin permeation enhancement, tack force, peel strength

## Abstract

The advent of skin patch formulation design and technology has enabled the commercialisation of methyl salicylate (MS) as a topical patch. However, the most fundamental aspect of skin permeation is unknown at present. The study aims to investigate the effect of solvent choice on the skin permeation of MS in a neat solvent system and patch formulation with an emphasis on patch adhesion. MS in six selected solvents (propylene glycol (PG), Transcutol^®^, isopropyl myristate, Labrasol^®^, Plurol^®^ oleique CC 497 and Maisine^®^ CC) was characterised and in vitro permeation studies were also performed. An ATR-FTIR analysis on solvent-treated skin was conudcted. Patch formulation was prepared and characterised for adhesion, in vitro drug release and skin permeation studies. The highest MS permeation was found in neat PG over 24 h (~90 μg/cm^2^) due to its strong skin protein conformation effect. Transcutol^®^ and isopropyl myristate showed better skin deposition and formulation retention, respectively. Nevertheless, PG enhanced the patch adhesion despite having a lower cumulative amount of MS permeated (~80 μg/cm^2^) as compared with Transcutol^®^ and Maisine^®^ (~110–150 μg/cm^2^). These two solvents, however, demonstrated better skin deposition and formulation retention but a lower patch adhesion. The unpredictable influence of the solvent on patch adhesion highlights the importance of the trade-off between patch adhesion and skin permeation during formulation design.

## 1. Introduction

Percutaneous absorption can deliver drugs to the body or the skin, and this has become a popular route due to its convenience and user-friendly administration [1,2,3,4]. Given such advantages, topical administration can allow for better patient compliance and reduced dosing frequency, with the possibility of withdrawing immediately [5,6,7,8,9,10]. This route has been used to deliver drugs for the treatment of localised diseases, such as pain management, for example.

Since the first successful introduction of the topical methyl salicylate (MS) patch [11,12], this drug is now commonly available in over-the-counter products for relieving pain [13,14,15,16,17]. Our previous review has summarised the different strategies employed to deliver MS through the skin [18]. However, the formulation to deliver MS is still challenging due to its volatility (vapour pressure: 5.3 Pa) [19]. Therefore, patches and semisolid dosage forms such as cream and ointment remain the dominant products in the current market [18].

Patches are an occlusive dosage form and could be more suitable than other aforementioned dosage forms because of their good stability and ability to minimise drug volatility during storage. Out of the different types of patch designs available, the drug-in-adhesive (DIA) patch design is easier to produce: usually by a simple mixing procedure before coating on the backing liner [20,21]. As the name indicates, DIA allows for the incorporation of drugs in the adhesive layer sandwiching between the backing and release liners [20,22,23]. This design is also currently employed for manufacturing MS patches that are available in the current market, owing to its convenience and easy production. In contrast, a matrix-dispersion system with an adhesive layer surrounding the drug layer dispersed in a polymer matrix involves multiple manufacturing steps and a higher production cost [20,24]. Reservoir patch design is typically bulky with the drug being dispersed or dissolved in solution, semisolid or in a suspension, and has a tendency towards formulation leakage [24,25].

Even though MS has been successfully marketed as a patch since 2008 under the brand Salonpas^®^ [26], there remains a huge gap in the fundamental research work to fully understand the skin permeation of this compound. The use of passive permeation enhancement strategies, including the incorporation of solvents (usually known as skin penetration enhancers), is preferred and can be easily adapted to the conventional patch formulation [18,27,28,29,30,31].

Patch adhesion is of the utmost importance for the efficacy of the treatment [5,23]. For this reason, the use of pressure-sensitive adhesive (PSA) is critical to ensure bonding to surfaces with light pressure application and that the patch will adhere to the skin without leaving residuals [32]. Formulation studies have demonstrated that the addition of drugs and excipients can modify the adhesive properties of PSA. Cilurzo, Gennari and Minghetti [5] have previously found that the influence of a substance added to the adhesion (usually recorded as tack force, shear adhesion and peel strength) is difficult to predict, and the data available in the current literature are contradicting. This could be due to the possibly unique interactions between the substance added and PSA for a particular combination at the specific amount or ratio used. As well, the patch formulation is usually complex and the multiple interactions between different materials can have a huge impact on the adhesion.

Hence, this work aims to elucidate the effect of solvents on the permeation of MS in a simple solvent system. To realise the importance of patch formulation design, the influence of both drugs and solvents on skin permeation, as well as patch characterisation were also emphasised. A total of six solvents were chosen based on the different solvent groups and their physicochemical properties, including propylene glycol (PG), Transcutol^®^ P (TRC), isopropyl myristate (IPM), Labrasol^®^ (LA) or caprylocapropyl macrogol-8 glycerides, Plurol^®^ oleique CC 497 (PLU) or polyglyceryl-3 dioleate and Maisine^®^ CC (MAI) or glycerol monolinoleate. These solvents are also generally regarded as safe and have been previously used for skin patch formulations [4,33,34,35,36,37,38,39,40,41,42].

## 2. Materials and Methods

### 2.1. Materials

MS was purchased from Jiangsu Puyuan Chemical Co., Ltd., Jiangsu, China. Internal standard of MS (lot number: R107Q0) was purchased from US Pharmacopeia, North Bethesda, MD, USA. Nikasol TS-620 (metha-acrylic alkyl ester copolymer aqueous emulsion) (named as “Nikasol” from here onwards) was purchased from Nippon Carbide Industries, Tokyo, Japan. Durabond PC 1879A (aqueous acrylic polymer dispersion) (named as “Durabond” from here onwards) was purchased from H.B. Fuller, Saint Paul, MN, USA. Polyvinyl pyrrolidone (PVP) K-90 (Kovidone^TM^) (USP grade, Molecular weight: 360,000) was purchased from Boai NKY Pharmaceuticals Ltd., Tianjin, China. Carboxymethyl cellulose (CMC) 4000 PA 07 (food grade) was purchased from Dow Chemical Company, Lansing, MI, USA. Anhydrous tartaric acid was purchased from Changmao Biochemical Engineering Co Ltd., Jiangsu, China.

D-sorbitol solution (70%) and PG (USP grade) were purchased from Biofresh Green Sdn Bhd, Prai, Malaysia. Tween 80 was purchased from Guangdong Huana Chemistry Co. Ltd., Guangdong, China. Methanol (High-Performance Liquid Chromatography, HPLC grade) was purchased from Thermo Fisher Scientific, Boston, MA, USA. TRC, MAI, LA and PLU were generous gifts by Gattefossé, Saint-Priest, France. IPM (USP grade) was purchased from KLK Oleo, Selangor, Malaysia. Trifluoroacetic acid for synthesis (≥99% purity) was purchased from Merck KGaA, Darmstadt, Germany.

Phosphate buffered saline (PBS) tablets (Dulbecco A; pH 7.3 ± 0.2 at 25 °C) was purchased from Oxoid Limited, Basingstoke, England. High vacuum grease was obtained from Dow Corning, Lansing, MI, USA. Trypsin (extracted from bovine pancreas, BAEE > unit/mg) was purchased from Macklin, Shanghai, China. Sodium bromide (purity > 98%) was purchased from R&M Chemicals, Essex, UK. The backing liner of patches (made of 100% polyethylene) was purchased from Wujiang City Lt Textile Co., Ltd., Jiangsu, China. The release liner of patches (polyethylene plastic) was purchased from Pikulthong Plastic Co., Ltd., Nakornpathom, Thailand. Aluminium foil bags were purchased from Imperial Packaging And Plastics Sdn Bhd, Selangor, Malaysia. Nylon membranes (pore size: 0.45 µm) were purchased from Gelman Sciences, Lansing, MI, USA.

Fresh porcine ears were obtained from a local abattoir (Penang, Malaysia). The porcine ear skin was cautiously removed from the underlying cartilage using a scalpel and washed with distilled water. Hairs and excess fats were trimmed with scissors and the full-thickness skin membranes were stored at −20 °C.

The SC isolation was conducted by trypsinisation of porcine ear skin using 0.2% *w*/*v* of trypsin in PBS at 37 ± 1 °C for 24 h [43]. After that, the SC sheets were separated and rinsed with distilled water. Before relevant analysis, the SC sheets were hydrated in a closed vessel above 27% *w*/*v* of sodium bromide solution in PBS overnight at room temperature [44]. The changes in the weight of SC sheets were monitored using analytical balance to obtain 20% water content in SC sheets [45].

### 2.2. Methods

#### 2.2.1. Solvent Selection

##### 2.2.1.1. Miscibility and Saturated Solubility Studies of MS

A miscibility study was conducted by adding 0.1 mL of MS to 0.9 mL of solvents and observed for phase separation over 24 h. An excess amount of MS was added to 3 mL of water and PBS with continuous stirring at 34.5 ± 0.5 °C for 24 h to study the saturated solubility of MS.

##### 2.2.1.2. ATR-FTIR Spectroscopy

The hydrated SC was fully immersed in excess MS or solvents for 24 h at 32 ± 0.5 °C. The treated SC sheets were then dried between two pieces of filter paper until the sheets did not wet the papers anymore [44]. A Shimadzu Prestige 21 spectrometer (Shimadzu Corp, Kyoto, Japan) coupled with a diamond ATR accessory (GladiATR, Pike Technologies, Madison, WI, USA) was used to record ATR-FTIR spectra for solvents, the untreated SC sheet (control) and the SC sheets treated with MS and solvents. The spectra were recorded over a wavenumber range of 4000–600 cm^−1^ with a resolution of 4 cm^−1^ and a total of 32 scans. The spectra obtained were analysed and presented using Origin Pro 2019 (version 9.6) software (Northampton, MA, USA).

##### 2.2.1.3. In Vitro Permeation and Mass Balance Studies

In vitro permeation experiments were performed using Franz-type diffusion cells with a diffusion area of about 0.79 cm^2^ [46,47]. A full thickness of porcine ear skin was placed between the donor and receptor compartments by having the SC facing the donor compartment. The receptor compartment was filled with roughly 3 mL of degassed PBS. The Franz cells were thermostated at 34.5 ± 0.5 °C in a water bath to maintain the normal skin surface temperature (32.0 ± 0.5 °C) with continuous stirring. A finite dose (10 µL/cm^2^) of MS (10% *v*/*v*) in the respective solvent was placed onto the skin surface with occlusion. A volume of 200 µL of PBS was withdrawn from the receptor compartment at 15 and 30 min as well as 1, 2, 4, 6, 8, 12 and 24 h. After the permeation studies, the skin surface was washed with methanol (0.5 mL × 2) (termed as washing) and the skin was then cut to soak in 1 mL of methanol for extraction of the drug in the skin (termed as extraction) [46,48]. Both samples were mixed overnight and analysed by HPLC. The experiment was conducted in triplicate.

##### 2.2.1.4. HPLC Analysis

Samples were analysed using a liquid chromatographic system (Shimadzu, Kyoto, Japan) equipped with a CBM-20A system controller connected to an LC-20AD solvent delivery unit, DGU-20A3 degasser system, CTO-10ASVP column oven, SIL-20AHT autosampler and SPD-20A ultraviolet-visible detector. The isocratic mobile phase used was methanol and 0.1% of trifluoroacetic acid in distilled water at a volume ratio of 85:15 and a flow rate of 1 mL/min. The mobile phase was filtered through a 0.45 µm nylon membrane filter under a vacuum condition and sonicated before use. A Phenomenex^®^ Luna C18 column (250 × 4.6 mm ID, 5 μm; Torrance, CA, USA) fitted with a Thermo Scientific Uniguard™ guard column (Waltham, Boston, MA, USA) with an ODS C18 cartridge (10 × 4 mm ID, 5 μm) was used as stationary phase. UV detection was set at 305 nm and a sample injection volume of 40 μL was used with a column temperature of 40 °C. An internal standard of MS was used during method development and validation. The retention time of MS was ~4.6 min. The limits of detection and quantification for MS were 0.08 and 0.25 µg/mL for a low drug concentration range of 0.25–7 µg/mL, respectively, as well as 4.20 and 12.72 µg/mL for a high drug concentration range of 5–150 µg/mL, respectively.

### 2.3. Patch Formulation

#### 2.3.1. Preparation of Patches

The patches were prepared according to the composition stated in Table 1. The composition of the materials used was optimised in preliminary work for acceptable adhesion and patch formation ability (data not shown).

Tartaric acid was dissolved in distilled water. After that, CMC and PVP K-90 were added to the solution and mixed at 40 °C until fully dissolved. After the mixture was cooled to room temperature, MS, Tween 80, D-sorbitol solution (5% *w*/*w*), Durabond and Nikasol, either composed or not composed by 5% of solvents, were then added to form a smooth white paste. The white paste was coated on the backing liner using a customised coater with a gap of 0.4 mm. The patches were dried at 50 ± 1 °C for 15 min. Lastly, the patches were attached to release liners and sealed in aluminium foil bags to be kept in a desiccator for at least a week before further studies.

The patches containing a solvent and drug were named according to the solvent abbreviation and followed by ‘P’ for the patch. For example, PG-P means PG was incorporated in the patch. Similar patches without drugs were also prepared and coded in a similar way; however, their names ended with ‘SP’, such as ‘PG-SP’. Drug- or/and solvent-free patches (control) were coded as MS-P (patches without solvent) and EP (patches without solvent and drug).

#### 2.3.2. Characterisation of Patches

##### 2.3.2.1. Weight of Patches

Ten patches with a size of 3 × 3 cm^2^ were cut before removing the release liner. The weight of the DIA matrix was determined by deducting the weight of the same-sized backing liner.

##### 2.3.2.2. Thickness of Patches

The patch thickness (after removal of release liners) was measured using a Mitutoyo Digimatic micrometre at 10 different locations. The thickness of the DIA matrix was determined by subtracting the thickness of the patch from the thickness of backing liner.

##### 2.3.2.3. Drug Content

Drug-containing patches were cut into a size of 1 × 1 cm^2^ and transferred into 2 mL of methanol. The samples were shaken overnight before HPLC analysis. The study was performed in triplicate.

##### 2.3.2.4. ATR-FTIR Spectroscopy of Patch Formulation

The patches were analysed by ATR-FTIR spectroscopy and presented in the same manner as mentioned in Section 2.2.1.2.

##### 2.3.2.5. Tack Force

The measurement of tack force was performed using a TA-XT plus Texture Analyser (Stable Micro System, Godalming, UK) equipped with a 30 kg load cell which was adapted from the work by Musazzi et al. [49]. The patch was cut into 2.5 × 6 cm^2^ and fixed on the platform of the Texture Analyser with 1 kg roller. A stainless-steel cylindrical probe (diameter: 1 cm, area of probe: 0.79 cm^2^) was lowered down to the patch at 0.1 mm/s and pressed with a force of 0.05 N for 5 s after contacting the patch. The probe was then removed at a debonding rate of 0.1 mm/s. The test was done in three replicates. The maximum adhesion peak force was obtained to calculate the tack force using the equation

(1) $$\mathrm{Tack}\;\mathrm{force}\;(N/{\mathrm{cm}}^{2})=\frac{\mathrm{Maximum}\;\mathrm{force}\;\left(N\right)}{\mathrm{Contact}\;\mathrm{area}\;\mathrm{of}\;\mathrm{probe}\;\left({\mathrm{cm}}^{2}\right)}$$

##### 2.3.2.6. Peel Strength

The 180° peel adhesion test was carried out at room temperature by using the same Texture Analyser as mentioned in Section 2.3.2.5. The patch was cut into a size of 25 × 2.5 cm^2^ and applied onto a piece of porcine ear skin with three-time rolling using a 1 kg roller. The patch was pulled at 180° from the substrate at a constant rate of 2 mm/s. This study was performed in triplicate. The highest peak force was obtained and the peel strength was calculated using the equation

(2) $$\mathrm{Peel}\;\mathrm{strength}\;(N/\mathrm{cm})=\frac{\mathrm{Peak}\;\mathrm{peeling}\;\mathrm{force}\;\left(N\right)\;}{\mathrm{Width}\;\left(\mathrm{cm}\right)}$$

##### 2.3.2.7. In Vitro Release Studies

In vitro release studies were carried out using Franz diffusion cells as described in Section 2.2.1.3, but using a nylon membrane instead of porcine ear skin. A patch was trimmed according to the area of the donor to be applied to the membrane. The patch was adhered to the membrane using a 0.5 kg roller before mounting between the donor and the receptor. The donor was occluded with parafilm to reduce MS evaporation. The samples were withdrawn at 5, 15, 30 and 45 min and 1, 2, 4, 6, 8, 12 and 24 h from the receptor chamber. This test was conducted in triplicate. The data were fitted into different drug release kinetic models including zero order, first order, Higuchi and Korsmeyer–Peppas models [50,51]. The coefficient correlation (*R^2^*) and release exponent (*n*) were calculated to determine the best-fitting model.

##### 2.3.2.8. In Vitro Permeation and Mass Balance Studies

The in vitro permeation study was carried out as discussed in Section 2.2.1.3 using porcine ear skin without occlusion. The patch was cut according to the area of the donor and applied on the porcine skin before assembling in the same manner as mentioned in Section 2.2.1.3. The same condition and time intervals for sample collection were performed accordingly, as mentioned in Section 2.2.1.3. A mass balance study was also conducted using the same procedure stated in Section 2.2.1.3. The patch in the donor chamber was removed and soaked in methanol used for skin surface washing (0.5 mL × 2). The skin membrane was cut and soaked in 1 mL of methanol. The samples were left mixing overnight before HPLC analysis.

##### 2.3.2.9. Statistics

All of the statistical analyses were analysed using *p* < 0.05 as the minimum level of significance in all cases. The results are expressed as mean with standard deviation (SD). All data showed normal distribution with skewness and kurtosis values less than 1 (or more than −1). The parametric data were analysed using one-way ANOVA with post-hoc Tukey’s test.

## 3. Results and Discussion

### 3.1. Solvent Selection

#### 3.1.1. Miscibility and Saturated Solubility Studies of MS

There was no phase separation observed for all six solvents to which 10% *v*/*v* of MS was added. Detailed observations are provided in the Supplementary Information (Table S1). The saturated solubility of MS in PBS and water at 32 °C over 24 h was 2.9 ± 0.2 and 3.4 ± 0.5 g/L, respectively. Previously, the aqueous solubility of MS was only 0.64 g/L at 21 °C, as reported by Yalkowsky and Yan [52]. The solubility obtained here was higher because of the higher temperature used. The solubility data (>10 mg/L) are sufficient to maintain the sink condition for in vitro permeation studies [53].

#### 3.1.2. ATR-FTIR Spectroscopy

**Figure 1:**
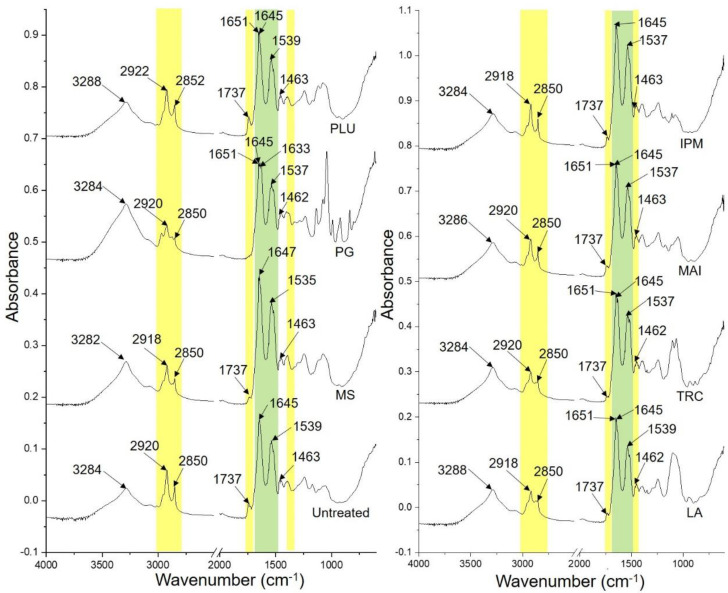
Comparisons of ATR−FTIR spectra of untreated porcine SC sheets and those treated with MS and solvents, with modifications highlighted in the lipid regions (yellow bar) and protein regions (green bars). MS: methyl salicylate; PG: propylene glycol; PLU: Plurol^®^ oleique CC 497; LA: Labrasol^®^; TRC: Transcutol^®^ P; MAI: Maisine^®^ CC; IPM: isopropyl myristate.

The ATR-FTIR spectra of hydrated SC sheets treated with MS and solvents for 24 h were compared with the untreated hydrated SC sheet as presented in Figure 1.

Table 2 shows the frequency band signals of ATR-FTIR spectra observed for the control porcine SC sheets and those treated with MS and solvents based on the related SC components. There were two critical changes detected for porcine SC sheets treated with MS and solvents: the CH_2_ asymmetric stretching vibrations for lipids and amide I band for protein.

The SC sheets treated with MS, LA and IPM induced a red shift in the CH_2_ asymmetric stretching vibrations from 2920 cm^−1^ to 2918 cm^−1^. However, a blue shift in the same and related lipid peaks was observed in rat SC treated with MS, which was ascribed to the structural disturbance in the SC lipids [15]. Mansour et al. [54] and Casiraghi et al. [55] also reported red shift for the same lipid peaks in human SC treated with IPM. Nevertheless, the red shift for this lipid peak has been related to SC lipid disordering [56]. Such a mechanism has also been proposed in thermal analysis using differential scanning calorimetry (DSC) with a reduced transition temperature for lipid-related endotherms (T_2_ and T_3_) of human SC [43,57,58,59,60]. Although no current data are available for LA, it is speculated that LA causes the SC lipid disordering similarly to IPM and MS, due to a similar shift in the CH_2_ asymmetric stretching.

PLU is the only solvent that showed a shift of both CH_2_ asymmetric and symmetric stretching vibrations to a higher frequency (Table 2). Yang, Liu, Quan and Fang [42] observed a similar shift in the same vibration (from 2918 cm^−1^ to 2922 cm^−1^) when analysing rat skin treated with PLU using ATR-FTIR spectroscopy, suggesting interactions with the SC intercellular lipids which reduce the diffusional resistance. Salimi et al. [61] proposed SC lipid disruption and fluidisation by PLU based on shifts in the CH_2_ asymmetric (from 2948 cm^−1^ to 2937 cm^−1^) and symmetric (from 2822 cm^−1^ to 2895 cm^−1^) stretching vibrations in rat SC, probably due to PLU acting as a surfactant and solubilising the SC lipids. For the rest of the solvents (PG, TRC and MAI), no change was detected for the lipid-related peaks.

Interestingly, we also noticed the absence of the C=O stretching vibration for lipids near 1737 cm^−1^ for the PG-treated SC sample. This indicates that the lipid polar regions may be fully disturbed by PG [62]. Also, a minor red shift in the CH_2_ scissoring vibrations for the SC sheets treated with PG, LA and TRC was noticed, suggesting a possible change in the lateral packing of the SC lipids [63].

On the other hand, changes in the protein peak were also recorded. All of the investigated solvents showed either blue shift (MS only) or peak splitting for the amide I band except for IPM, which showed no change. IPM is known to cause a lipid fluidising effect but is less likely to interact with the SC protein in DSC [58,64,65] and neutron diffraction analyses [66]. This explains the unchanged protein peaks in the results for the SC treated with IPM.

For MS, a minor blue shift was previously observed by Wang, Zhao, Chen, Li, Hao, Li, Mei, Lan and Wu [15] from 1651 to 1652 cm^−1^ for rat SC treated with MS, which is different from the current study. Nevertheless, the shift to lower or higher wavenumbers for this amide I peak is strongly related to the protein conformation change [67].

The SC sheet treated with PG showed two new peaks at the amide I band (1651 and 1633 cm^−1^). Such changes have been shown as evidence in other studies that PG may change the SC protein conformation (keratin) from α-helices to random coils and β-sheets [68,69]. The effect of PG on both human and porcine SC has been studied using thermal analysis (DSC and nano-thermal analysis) and found to be related to the SC protein dehydration that may weaken the skin barrier function [43,70]. Shah et al. [71] also suggested that PG resulted in the loss of the SC protein peak (120 °C) in the thermogravimetric analysis of rat SC. Besides, they also observed new peaks at 1648, 1642 and 1632 cm^−1^ in the amide I band, which represented the conversion of α-helices (disappearance of peak at 1656 cm^−1^) to random coil and β-sheets during FTIR analysis.

Instead of splitting into three peaks, the amide I band for the SC sheets treated with PLU, LA, TRC and MAI showed only two peaks at 1651 and 1645 cm^−1^. This suggests a change in the protein conformation to random coil structures [68,69]. These observations have not been widely reported; only the changes in the lipids were reported in the literature for these solvents using rat skin, as discussed previously [41,61,72]. TRC was previously observed to increase the mobility of the SC keratin filament terminals due to the dehydration effect of the solvent [73,74].

The shortfall of the related studies in the current literature is the use of animal skin other than human or porcine skin to evaluate the mechanism action of solvents on the SC. We acknowledge the importance of referring to the literature reporting on human or porcine skin which is widely known as a human skin surrogate due to a similar physiological and anatomical structure [75,76]. However, limited relevant studies were available, especially using suitable skin models. In addition, other methods of analysis used for evaluation are also included for discussion here, especially PLU, LA and MAI.

#### 3.1.3. In Vitro Permeation and Mass Balance Studies

**Figure 2:**
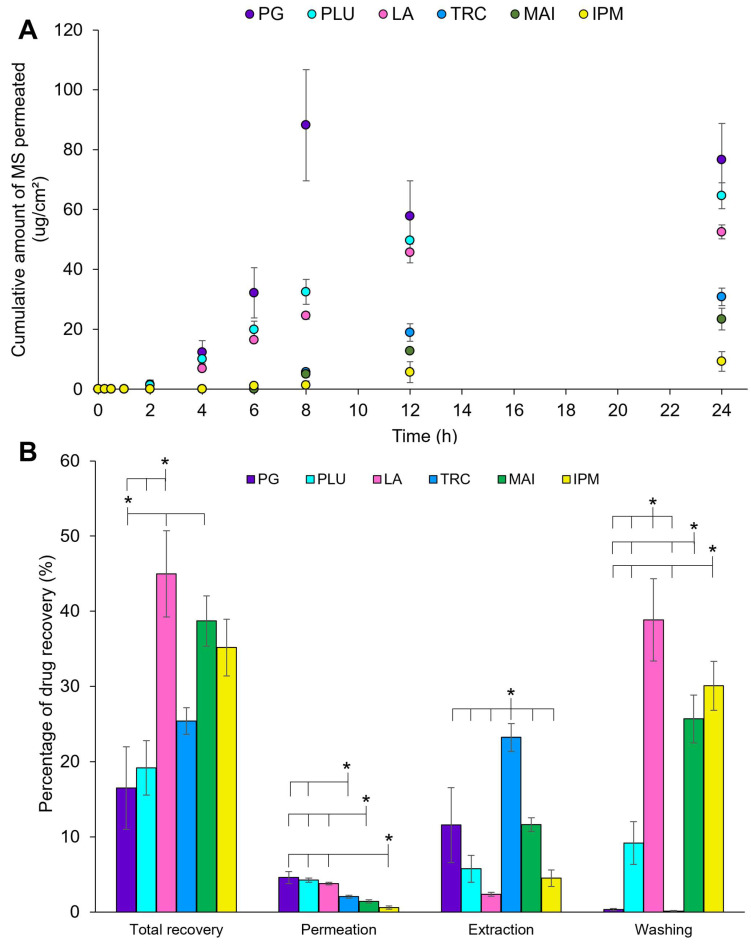
(**A**) In vitro permeation and (**B**) mass balance studies of 10% *v*/*v* of MS in neat solvents (PG: propylene glycol; PLU: Plurol^®^ oleique CC 497; LA: Labrasol^®^; TRC: Transcutol^®^ P; MAI: Maisine^®^ CC; IPM: isopropyl myristate) for finite dose (10 µL/cm^2^) with occlusion over 24 h using porcine ear skin (n = 3, mean ± SD; * *p* < 0.05, ANOVA).

Figure 2 represents the in vitro permeation profiles and mass balance studies of MS in neat solvents using porcine ear skin over 24 h with occlusion.

Overall, PG showed the highest cumulative amount of MS permeated over 24 h among all solvents, peaking at 8 h with 88.2 ± 32.2 µg/cm^2^ of MS permeated (Figure 2A). This is followed by PLU and LA, showing a cumulative amount of MS permeated as 65.3 ± 7.5 and 52.5 ± 4 µg/cm^2^, respectively, at 24 h. On the other hand, TRC, MAI and IPM demonstrated much lower MS permeation over 24 h with a longer lag time (~6 h). The cumulative amount of MS permeated was 2.9–9.5-fold lower than PG, recording at 30.8 ± 5.1, 23.4 ± 6.3 and 9.3 ± 5.7 µg/cm^2^, respectively.

Based on the ATR-FTIR analysis in the previous section, these solvents changed the SC lipids and/or protein conformation for permeation enhancement. In particular, the highest drug permeation by PG could be related to a greater effect of protein conformation change, with a total conversion into the β-sheet arrangement and random coil structure as compared to PLU and LA. Besides, PG is known to be depleted from the formulation due to rapid skin diffusion and solvent evaporation as a result of its small molecular size and a high vapour pressure [34,46,77,78]. This could promote the increased thermodynamic activity of MS, leaving a highly concentrated drug solution on the skin surface and maximising drug permeation, especially at 8 h [28,79]. Following this, a reduced MS permeation may be because of the loss of volatile MS and the depletion of PG.

Besides the effect on the SC components, PLU and LA may serve as surfactants by forming micelles with MS and water to increase the solvation of MS within the SC in order to travel across the skin [80,81]. This could be the possible reason for a better MS permeation than TRC, MAI and IPM.

The results of mass balance studies are displayed in Figure 2B. The total recovery of MS was generally less than 50% because of the high volatility of MS. LA, IPM and MAI exhibited among the highest total recovery of MS (~40–45%); however, they were not statistically different (*p* > 0.05, ANOVA). The high recovery was mainly contributed to by the remaining solvents (washing). This could indicate the ability of solvents to reduce the loss of the volatile drug and to retain in the drug solution, particularly for IPM and MAI. IPM was previously found to minimise the volatility of ethanol by showing an improved weight loss of a gel formulation from 33.2% to 20.9% as a result of a lower vapour pressure of IPM (0.00033 kPa) than ethanol (6.0 kPa) [82]. This could also be the reason for the low permeation using these two solvents.

Even though TRC has a low MS permeation, the skin deposition (extraction) was the highest (23.2 ± 3.2%) (*p* < 0.05). This is followed by MAI (11.6 ± 1.6%) and PG (11.6 ± 8.7%). TRC has been shown to form a skin depot by retaining drugs in the skin, especially for lipophilic drugs such as MS, and this may increase the lag time [83,84]. A higher human skin deposition for lipophilic compounds was seen in simple drug solutions in TRC than PG including kahalalide F (TRC: ~0.2%; PG: ~0.07–0.1%) [85], and anthramycin (TRC: 8.5%; PG: 2.1%) [86]. The presence of TRC also enhanced the human skin deposition of petrolatum in simple solvent systems from 83.1 ± 8.8% to 88 ± 8.6% [87].

The total recovery of MS for PG was the lowest (~16%) among all solvents used. This could be related to the aforementioned phenomenon of PG depletion.

### 3.2. Patch Formulation

Patches were successfully formed with only PG, IPM, TRC and MAI but not with PLU and LA due to the presence of an oily surface for PLU-P and total loss of adhesiveness for LA-P (Figure S1 in the Supplementary Information section). Therefore, these two patches were not selected for further characterisation.

#### 3.2.1. Characterisation of Patches

##### 3.2.1.1. Patch Weight, Thickness and Drug Content

The weight and thickness of patches as well as drug content are demonstrated in Table 3. The DIA matrix has a weight of 6–7 mg/cm^2^ and a thickness of ~0.09 mm with ~2.3–2.5 mg/cm^2^ of MS from 10% of MS loading. There is no significant difference in the weight, thickness and drug content of patches, which indicates the consistency of the patch coating process (*p* > 0.05, ANOVA).

##### 3.2.1.2. ATR-FTIR Spectroscopy of Patches

**Figure 3:**
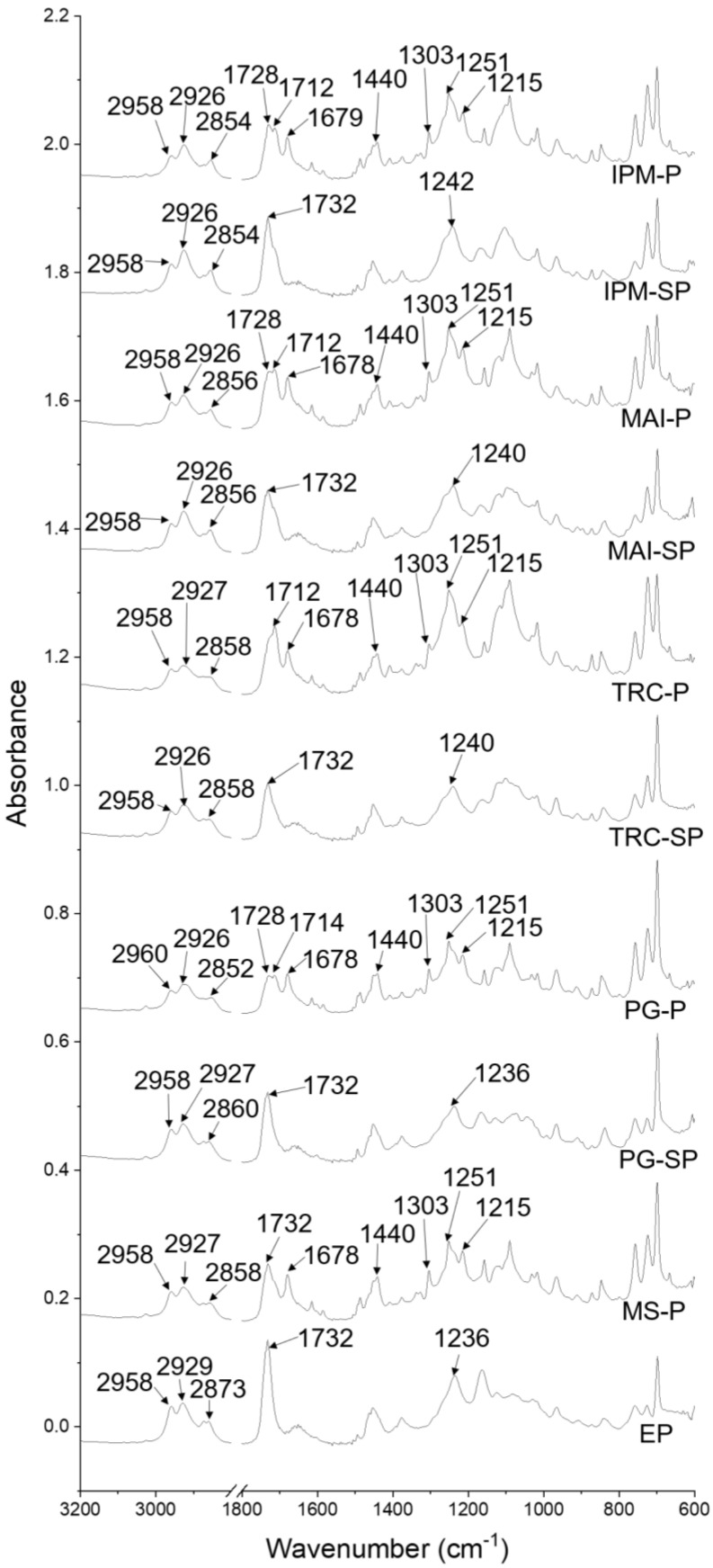
ATR−FTIR spectra of patches formed including EP (without solvent and drug), MS-P (with drug but without solvent), -SP (with solvent only) and -P (with drug and solvent). PG: propylene glycol; TRC: Transcutol^®^ P; MAI: Maisine^®^ CC; IPM: isopropyl myristate.

The ATR-FTIR spectra for the patches prepared are displayed in Figure 3 with the major absorption bands listed in Table 4. FTIR spectra with an enlarged region at 1200–1000 cm^−1^ can be found in Figure S2 (Supplementary Information).

After the addition of MS, there were four absorption bands recorded at 1678, 1303 and 1089 cm^−1^, reflecting the presence of MS (1674, 1301 and 1089 cm^−1^). The possible interactions between the MS and polymeric matrix may be postulated in a new absorption band at 1251 cm^−1^. A shift in the C-O and C-N stretching vibrations was seen in MS-P (1215, 1157, 1118, 1031 and 1016 cm^−1^) as compared with EP (1236, 1163, 1124, 1068, 1029 and 1018 cm^−1^).

When only solvents were incorporated (SP), the C=O stretching vibrations (PSA) at 1732 cm^−1^ remained unchanged. However, several changes were noted, as listed in Table 4, including blue shift or peak loss in the C-N stretching vibrations (TRC-SP, MAI-SP and IPM-SP), new peaks in the C-O stretching vibrations (TRC-SP and MAI-SP) and multiple peak shifts in the C-O stretching vibrations (PG-SP, MAI-SP and IPM-SP) as compared with EP. This may evidence the interactions of PSA with solvents.

When both MS and solvents were included in the patches, the three characteristic peaks for MS molecules remained unchanged for PG-P, TRC-P and MAI-P as compared with MS-P. However, the C=O stretching vibrations for MS were shifted from 1678 to 1679 cm^−1^ in IPM-P. The C=O stretching vibrations for PSA showed two absorption bands (1728 and 1714–1712 cm^−1^) in IPM-P, MAI-P and PG-P. At the same time, the ATR-FTIR spectrum of TRC-P showed a shift in the C=O stretching vibrations from 1732 to 1712 cm^−1^. The OH bending (1440 cm^−1^) and C-O (1251 and 1157 cm^−1^) and C-N (1215 cm^−1^) stretching vibrations also remained unchanged in the ATR-FTIR spectra for all patches containing MS and solvents.

The FTIR spectrum of EP showed the characteristic peaks of PSA (Durabond and Nikasol) and the polymeric matrices (PVP K-90 and CMC). These include the C=O stretching vibrations (PSA) at 1732 cm^−1^, C-O stretching vibrations (PSA) at 1163, 1124 and 1082 cm^−1^, C-N stretching vibrations (polymeric matrix) at 1236 and C-N stretching vibrations at 1068, 1029 and 1018 cm^−1^ (PSA).

There was a new peak corresponding to the C-O stretching vibrations observed in PG-P (1126 cm^−1^), TRC-P (1097 cm^−1^), MAI-P (1099 cm^−1^) and IPM-P (1101 cm^−1^) as compared with MS-P (1118 cm^−1^). The ATR-FTIR spectra of PG-P (1157 and 1118 cm^−1^), TRC-P (1157 and 1116 cm^−1^), MAI-P (1157 and 1116 cm^−1^) and IPM-P (1157 and 1121 cm^−1^) showed a shift in the C-O stretching vibrations as compared with MS-P (1157 and 1118 cm^−1^). The C-O stretching vibration for MS at 1089 cm^−1^ remained unchanged in all patches containing solvents and MS.

A new absorption band was seen in PG-P at 1043 cm^−1^, referring to the C-N stretching vibrations. The C-N stretching vibrations at 1031 and 1016 cm^−1^ remained unchanged in PG-P, TRC-P and IPM-P. MAI-P displayed a shift in the C-N stretching vibrations from 1031 to 1029 cm^−1^ while another absorption band (1016 cm^−1^) remained the same as MS-P.

##### 3.2.1.3. Tack Force

**Figure 4:**
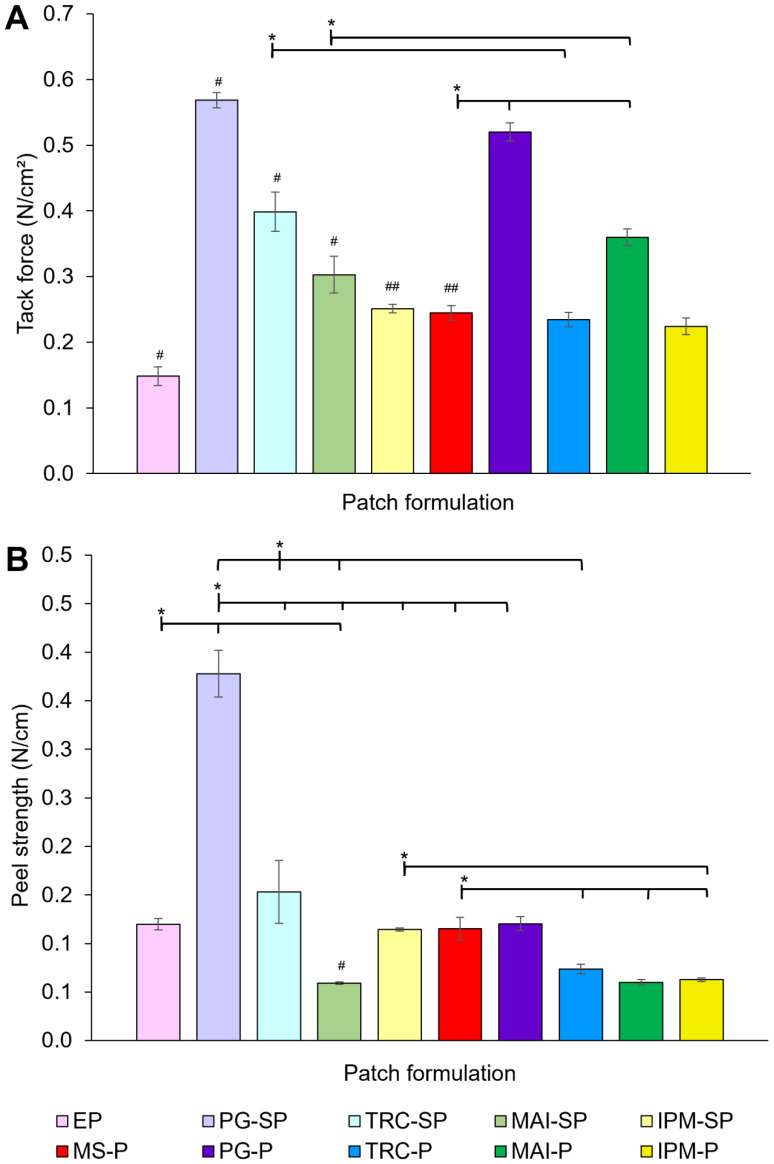
(**A**) Tack force and (**B**) peel strength of different patches prepared with and without solvent and/or drug (n = 3, mean ± SD; * *p* < 0.05, ANOVA; # statistically different comparing EP, -SP and MS-P; ## statistically different comparing EP, -SP and MS-P but not between the samples with the same symbols). PG: propylene glycol; TRC: Transcutol^®^ P; MAI: Maisine^®^ CC; IPM: isopropyl myristate; EP: patches without solvent and drug; MS-P: patches with drug but without solvent; -SP: patches with solvent only; -P: patches with drug and solvent.

The tack force of patches is presented in Figure 4A. EP exhibited a tack force of 0.15 ± 0.01 N/cm^2^. The addition of MS (MS-P) or solvents significantly increased the tack force (0.24 ± 0.01 N/cm^2^) (*p* < 0.05, ANOVA). The highest tack force was recorded for PG-SP (0.57 ± 0.01 N/cm^2^) (*p* < 0.05).

On the other hand, the incorporation of both solvents and MS caused a reduction in tack force of PG-P (0.23 ± 0.01 N/cm^2^), TRC-P (0.23 ± 0.01 N/cm^2^) and IPM-P (0.22 ± 0.01 N/cm^2^) when compared to the respective solvent patches; however, only the result for the TRC-P is statistically significant (*p* < 0.05). MAI enhanced the tack force of drug-loaded patches (0.36 ± 0.01 N/cm^2^) (*p* < 0.05).

In comparison to MS-P, PG-P and MAI-P showed a significant increase in the tack force (*p* < 0.05) but no difference was found for TRC-P and IPM-P (*p* > 0.05). This could indicate that TRC and IPM did not affect the tack force in the presence of MS. When compared with the corresponding solvent patches, all drug-loaded patches showed a significant decrease in the tack force except for MAI-P, which demonstrated an increased tack force (*p* < 0.05).

The incorporation of drugs and solvents in the formulation may demonstrate a plasticisation or anti-plasticisation effect on the tack force of patches, depending on the types and concentration of actives, polymers or other excipients [5,88]. Plasticisation usually reduces tack force and vice versa for anti-plasticisation.

A higher tack force in MS-P as compared with EP may be due to the anti-plasticisation effect of MS on PSA and the polymeric matrix. This may be indicated by several peak shifts of the C-N stretching vibrations as recorded in the FTIR analysis, which shows the interactions of PSA and polymer with MS for such an effect on tack force.

Solvents may also exert a similar anti-plasticisation effect to MS-P, and such a change can be implied by the influence of the solvents on the polymer matrix and PSA, as previously discussed with regard to the FTIR analysis. Four peaks related to the C-N stretching vibrations were observed in PG-SP (1236, 1043, 1031 and 1018 cm^−1^) and TRC-SP (1240, 1068, 1029 and 1018 cm^−1^), while MAI-SP (1240, 1029 and 1016 cm^−1^) and IPM-SP (1242, 1029 and 1016 cm^−1^) showed only three absorption bands. This may suggest a stronger anti-plasticisation effect by PG and TRC as compared with MAI and IPM.

Despite this, the combination of MS and solvents showed a different impact by having a plasticisation effect that decreased the tack force, except for MAI-P which showed a stronger anti-plasticising role. We observed the changes (additional peak or peak shift) in the C-N stretching vibrations (PSA) under the influence of both MS and solvents that contributed to the plasticisation action, while the vibration remained unchanged for MAI-P, suggesting the ability of the combined MS and MAI to enhance the anti-plasticising action.

Generally speaking, the effect of solvent and drug on the mechanical strength of patches is unpredictable [5]. Some of the materials used, including MS and solvents, are generally recognised as plasticisers for films and patches [89,90,91]; however, these materials exerted an anti-plasticisation effect here, which may be due to the interactions with PSA and polymer matrix as observed in the ATR-FTIR analysis. Previously, Wang, Ma, Liu, Han and Tang [17] reported the enhancement effect of plasticiser (liquid paraffin) on the tack force of MS patches. In addition, Tuntiyasawasdikul et al. [92] also observed an increased tack force of patches containing methoxyflavones with the addition of different solvents including IPM, menthol, N-methyl-2-pyrrolidone, PG and oleic acid.

The effect of solvents on the tack force of patches can be concentration-dependent [93,94]. The interplay between plasticiser and drug contents has been previously reported to affect the mechanical properties of polymeric films differently as a result of plasticising or anti-plasticising effects from the different material compositions [95,96,97,98]. It is speculated that the same idea could be applied in the current work

##### 3.2.1.4. Peel Strength

The measurement of peel strength as shown in Figure 4B was evaluated using porcine ear skin to mimic the real-life condition when applying patches. EP exhibited a peel strength of 0.12 ± 0.01 N/cm. The incorporation of MS did not show any significant effect on the peel strength of MS-P (0.12 ± 0.01 N/cm) (*p* > 0.05, ANOVA). A similar observation was reported for patches incorporated with certain solvents including TRC-SP (0.15 ± 0.03 N/cm) and IPM-SP (0.11 ± 0.002 N/cm). However, a significant increase in peeling strength was observed in PG-SP (0.38 ± 0.02 N/cm); MAI halved the peeling strength of patches (0.06 ± 0.001 N/cm) (*p* < 0.05).

When the solvent patches were added with MS, a significant reduction in peeling strength was only exhibited in PG-P (0.15 ± 0.03 N/cm), TRC-P (0.07 ± 0.01 N/cm) and IPM-P (0.06 ± 0.002 N/cm) as compared to the corresponding solvent patches (*p* < 0.05). MAI-P is the only drug-loaded patch that showed a similar peeling strength (0.06 ± 0.003 N/cm) to its solvent patch (*p* > 0.05), while only TRC-P, MAI-P and IMP-P showed a reduced peel strength as compared with MS-P.

The peel strength depends on how the polymeric matrix of patches contact and wet the skin surface (rate of wetting) simultaneously [5,99]. The hydrophilic–hydrophobic balance between patches and the skin is an important aspect in determining the adhesion of patches [100]. The adhesion of patches can be enhanced by reacting with or absorbing the water in the skin [101].

Hydrophilic solvents such as PG have low water resistance and may absorb water from the skin to promote interactions between patches and the skin and to maintain the interfacial hydrophilic–hydrophobic balance for better skin adhesion [102,103]. Oily solvents with a high water resistance, such as MAI, may leach out from the patch and accumulate at the skin–patch interface, which reduces the skin contact, leading to a lower peel strength. Thus, it is hypothesised that PG promoting better skin contact improved the peel strength of the solvent patch.

For the same reason, the addition of hydrophobic MS also reduced the peel strength of solvent patches, especially PG-P. A greater reduction in peel strength was noted in PG-P and TRC-P, which may justify the possible counteracting effect of MS on the addition of these solvents to the polymer matrix.

Unusual observations were detected where the peel strength for some patches showed an opposite effect to the tack force. This can be seen when MAI was used, where the tack force of patches increased but the peel strength decreased, for instance. Nevertheless, this phenomenon has been reported in the literature, as summarised in Table 5.

It must be emphasised that the properties of the surface to be adhered to by the patch are vital to allow interfacial interactions with the polymer matrix in the patch. The tack force measurement investigates the probe detachment profiles of patches after the interactions of the polymer matrix with the inert surface of the stainless-steel probe. We believe that such interactions could be predominantly governed by the plasticisation and anti-plasticisation effect of MS and solvents with polymers in the formulation discussed earlier, while the peel strength measured on organic materials such skin may purely rely on the influence of the interfacial hydrophilic–hydrophobic balance. Therefore, the different surface interactions suggested here may have some difficulty in exactly predicting the mechanical properties of patches.

##### 3.2.1.5. In Vitro Drug Release Studies

**Figure 5:**
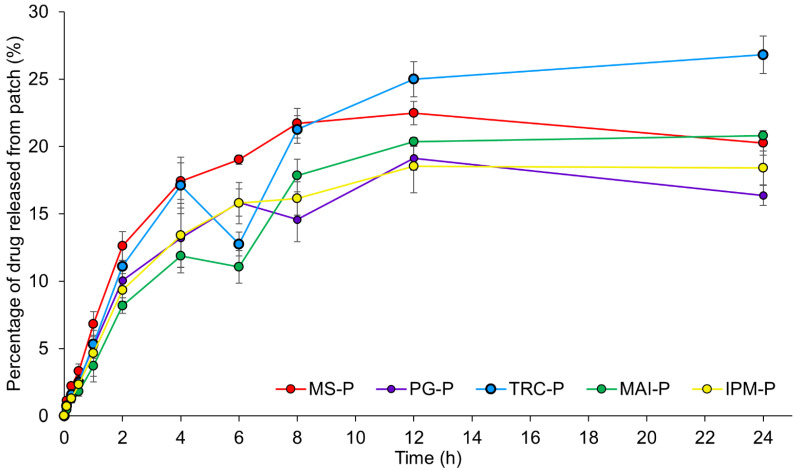
In vitro release studies of MS-loaded patches with different solvents (PG: propylene glycol; TRC: Transcutol^®^ P; MAI: Maisine^®^ CC; IPM: isopropyl myristate) (n = 3, mean ± SD). MS: methyl salicylate; MS-P: patches with drug but without solvent; -P: patches with drug and solvent.

Figure 5 represents in vitro drug release studies using MS-loaded patches over 24 h. The release of MS from MS-P reaches the maximum at 12 h (22.5 ± 0.9%) before a decrease in drug release to 20.3 ± 0.9% at 24 h, probably due to the loss of volatile MS. With the aid of solvents, MS was released more slowly, except for TRC-P. TRC-P showed a steady increase in drug release over 24 h with a total of 26.8 ± 1.4% of MS released, which is higher than that achieved by MS-P. The rest of the patches generally showed a lower drug release from 4 h onwards with a final MS release ranging from ~16 to 21% at 24 h.

The drug release results were also fitted to different kinetic models, as summarised in Table 6. The release mechanism of topical patches in the current project best fits the Korsmeyer–Peppas model, with *R^2^* closer to 1. The drug release mechanism follows non-Fickian diffusion (*n* = 0.58–0.72), where drug release relies on the swelling of the polymeric matrix that can be affected by water diffusion and the flexibility of polymer chains [106].

##### 3.2.1.6. In Vitro Drug Permeation Studies

**Figure 6:**
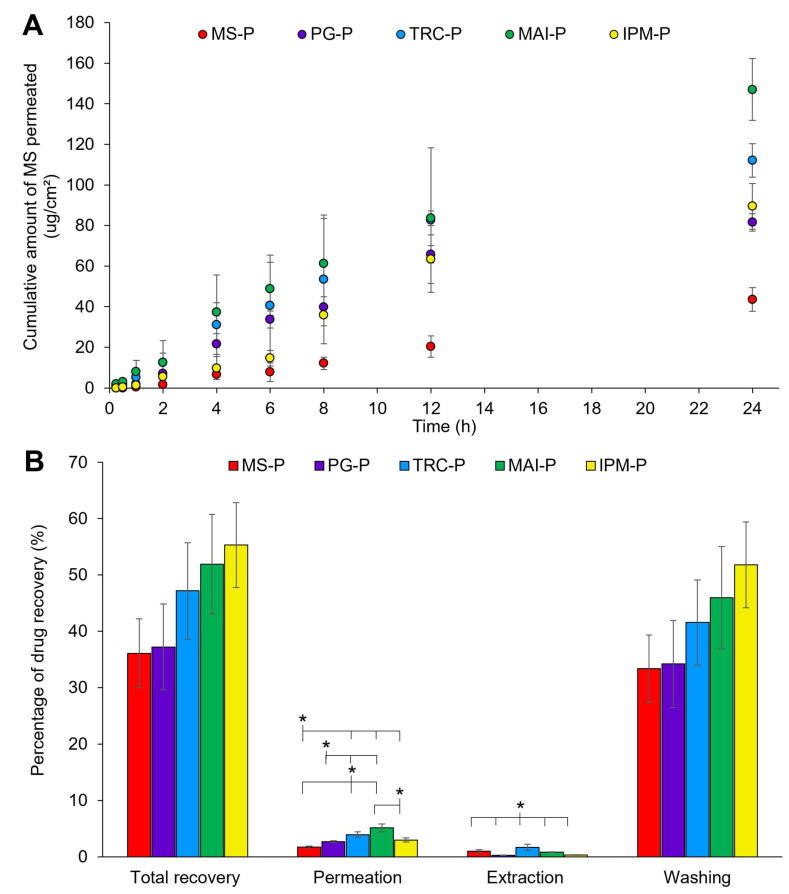
(**A**) In vitro permeation and (**B**) mass balance studies of MS-loaded patches with different solvents (n = 3, mean ± SD; * *p* < 0.05, ANOVA). PG: propylene glycol; TRC: Transcutol^®^ P; MAI: Maisine^®^ CC; IPM: isopropyl myristate) over 24 h using porcine ear skin. MS: methyl salicylate; MS-P: patches with drug but without solvent; -P: patches with drug and solvent.

Figure 6 demonstrates in vitro permeation and mass balance studies of patches using porcine ear skin over 24 h. Despite having a high drug release, MS-P only showed a low MS permeation through the skin (43.6 ± 5.9 µg/cm^2^) (Figure 6A). The addition of solvents generally showed a higher skin permeation of MS, signifying the role of solvents in enhancing permeation.

In the current study, MAI-P was found to have the highest cumulative amount of MS permeated at 24 h (147.0 ± 15.2 µg/cm^2^). The permeation of MS for TRC-P was comparable to the MAI-P, but achieved a lower cumulative amount of MS permeated at 24 h (112.1 ± 8.2 µg/cm^2^), while MS permeation from IPM-P and PG-P was lower (~80–90 µg/cm^2^) than the former patches with solvents.

Previously, TRC-P and PG-P showed a better drug release as compared with IPM-P (up to 12 h). Both patches accumulated a high MS content on the skin surface that increased the drug thermodynamic activity for enhanced permeation of MS [28,79]. However, PG depletion, as discussed previously, may promote the loss of MS with reduced drug thermodynamic activity and skin permeation. The low skin permeation enhancement effect by IPM may be due to a much lower drug release.

Surprisingly, the highest permeation of MS was obtained by MAI-P, although the drug release of MAI-P was lower than that of TRC-P. MAI may be very good in retaining the drug and reducing its volatility, as previously mentioned during the mass balance studies of neat solvents. With the ability of MAI to interact with the SC components in decreasing the skin barrier function, these factors promoted the skin permeation of MS by MAI from the patch.

The total recovery of MS improved from ~42% in the neat solvent systems to ~55% in the patch formulation (Figure 6B) (*p* > 0.05, ANOVA). The patch formulation is crucial to reduce the loss of MS. Overall, MS-P exhibited the lowest drug recovery (36.1 ± 6.1%); however, the data are not statistically significant for the other samples (*p* > 0.05). Compared with the mass balance studies using neat solvents (Section 3.1.3), the total recovery of MS in patches followed the same trend: PG-P (37.2 ± 7.6%) < TRC-P (47.2 ± 8.6%) < MAI-P (51.9 ± 8.9%) < IPM-P (55.3 ± 7.6%). The volatility of TRC and PG may cause the depletion of MS in the patch formulation, leading to a low MS recovery. IPM and MAI showed a higher recovery of MS because of a higher affinity towards MS and the ability to retain MS in the patch.

Furthermore, the highest skin retention of MS (extraction) was still achieved by TRC-P (1.7 ± 0.6%) (*p* < 0.05). The same results were obtained in mass balance studies using neat drug solutions. It may be hypothesised that TRC retains MS in the skin and forms a depot [73,84,86,107,108].

## 4. Conclusions

Despite the MS patch having been marketed for more than a decade, the fundamental of skin permeation for this compound was not reported. This study shows the importance of solvent selection in enhancing MS permeation to the skin. In a simple neat solvent system, PG exhibited the strongest skin enhancement effect, which could be due to the greater effect of PG on the modification of protein conformation as compared with other solvents. Interestingly, LA, IPM and MAI were found to retain MS in the formulation and reduce the loss of this volatile drug. The highest skin deposition was obtained in the TRC solution due to its widely-reported skin depot effect. When MS was formulated into a patch, the interplay between the solvents and MS impacted differently on the adhesion studies. Even though PG gave the highest adhesion and skin permeation in the neat solvent system, the skin permeation from the MS patch with PG was not the highest. Nevertheless, the MS permeation from this combination was slightly lower than the patches formulated with TRC and MAI, having a much poorer adhesion. The trade-off between patch adhesion and skin permeation should be considered for the patch formulation of MS apart from the consideration of preserving the volatile drug in the formulation.

## Figures and Tables

**Table 1:** Formulation of patches.

Formulation	Composition (% *w*/*w*)			
	EP	MS-P	Solvent-SP	Solvent-P
MS	-	10	-	10
Solvents	-	-	5	5
Durabond	8			
Nikasol	7			
Tartaric acid	3			
D-sorbitol solution	5			
Tween 80	0.4			
CMC	3.5			
PVP K-90	3			
Distilled water	Add up to 100			

MS: Methyl salicylate; CMC: carboxymethyl cellulose; PVP: Polyvinyl pyrrolidone.

**Table 2:** FTIR spectral assignments of porcine SC sheets treated with MS and solvents.

SC Components	Tentative Assignment		Wavenumber (cm^−1^)						
		Untreated	MS	PG	PLU	LA	TRC	MAI	IPM
Protein	Amide I	1645	1647	1651, 1645, 1633	1651, 1645	1651, 1645	1651, 1645	1651, 1645	1645
	Amide II	1539	1535	1537	1539	1539	1537	1537	1537
Lipids	CH_2_ asym str	2920	2918	2920	2922	2918	2920	2920	2918
	CH_2_ sym str	2850	2850	2850	2852	2850	2850	2850	2850
	C=O str	1737	1737	-	1737	1737	1737	1737	1737
	CH_2_ scissoring	1463	1463	1462	1463	1462	1462	1463	1463

Asym: asymmetric; sym: symmetric; str: stretching; SC: stratum corneum; MS: methyl salicylate; PG: propylene glycol; PLU: Plurol^®^ oleique CC 497; LA: Labrasol^®^; TRC: Transcutol^®^ P; MAI: Maisine^®^ CC; IPM: isopropyl myristate.

**Table 3:** Patch weight, thickness (n = 10, mean ± SD) and drug content (n = 3, mean ± SD) for different patches prepared with and without solvent and/or drug (PG: propylene glycol; TRC: Transcutol^®^ P; MAI: Maisine^®^ CC; IPM: isopropyl myristate; EP: patches without solvent and drug; MS-P: patches with drug but without solvent; -SP: patches with solvent only; -P: patches with drug and solvent).

Type of Patches	Weight * (mg/cm^2^)	Thickness * (mm)	Amount of MS (mg/cm^2^)
EP	6.3 ± 0.3	0.087 ± 0.002	-
PG-SP	6.9 ± 0.7	0.085 ± 0.003	-
TRC-SP	6.2 ± 0.8	0.087 ± 0.002	-
MAI-SP	6.8 ± 0.5	0.087 ± 0.004	-
IPM-SP	6.5 ± 0.6	0.086 ± 0.003	-
MS-P	7.0 ± 0.5	0.087 ± 0.004	2.5 ± 0.1
PG-P	6.8 ± 0.6	0.084 ± 0.003	2.3 ± 0.1
TRC-P	6.4 ± 0.7	0.086 ± 0.003	2.4 ± 0.1
MAI-P	6.7 ± 0.6	0.087 ± 0.003	2.4 ± 0.2
IPM-P	6.4 ± 0.6	0.086 ± 0.004	2.4 ± 0.1

* Drug−in adhesive matrix only.

**Table 4:** FTIR spectral assignments of different patches prepared with and without solvent and/or drug.

Tentative Assignment	Materials	Wavenumber (cm^−1^)									
		EP	MS-P	PG-SP	PG-P	TRC-SP	TRC-P	MAI-SP	MAI-P	IPM-SP	IPM-P
CH_3_ asym str	MS, polymeric matrix and PSA	2958	2958	2958	2960	2958	2958	2958	2958	2958	2958
CH_2_ asym str	Polymeric matrix and PSA	2929	2927	2927	2926	2926	2927	2926	2926	2926	2926
CH_2_ sym str	PSA	2873	2858	2860	2852	2858	2858	2856	2854	2854	2854
C=O str	PSA	1732	1732	1732	1728, 1714	1732	1712	1732	1728, 1712	1732	1728, 1712
C=O str	MS	-	1678	-	1678	-	1678	-	1678	-	1679
O-H bending	MS	-	1440	-	1440	-	1440	-	1440	-	1440
C-O str	MS	-	1303	-	1303	-	1303	-	1303	-	1303
C-O str	Polymeric matrix	-	1251	-	1251	-	1251	-	1251	-	1251
C-N str	Polymeric matrix	1236	1215	1236	1215	1240	1215	1240	1215	1242	1215
C-O str	PSA	1163, 1124, 1082	1157, 1118, 1089	1165, 1128, 1072	1157, 1126, 1118, 1089	1161, 1116, 1101, 1083	1157, 1116, 1097, 1089	1166, 1118, 1097, 1085, 1072	1157, 1116, 1099, 1089	1166, 1103, 1085	1157, 1121, 1101, 1089
C-N str	PSA	1068, 1029, 1018	1031, 1016	1043, 1031, 1018	1043, 1031, 1016	1068, 1029, 1018	1031, 1016	1029, 1016	1029, 1016	1029, 1016	1031, 1016

Asym: asymmetric; sym: symmetric; str: stretching; PG: propylene glycol; TRC: Transcutol^®^ P; MAI: Maisine^®^ CC; IPM: isopropyl myristate; EP: patches without solvent and drug; MS-P: patches with drug but without solvent; -SP: patches with solvent only; -P: patches with drug and solvent.

**Table 5:** Tack force and peel strength of patches with different formulations (CMC: carboxymethyl cellulose; IPM: isopropyl myristate; PVP: polyvinyl pyrrolidone; PG: propylene glycol.).

Formulation	Ingredient	Concentration (% *w*/*w*, Otherwise Stated)	Tack Force (N/cm^2^, Otherwise Stated)	Peel Strength (N/cm, Otherwise Stated)	Test & Substrate Used	Reference
Benztropine patch (DURO-TAK^®^ 2525)	Benztropine	5	1358 ± 35 g	980 ± 36 12.5 mm of patch length	Tack force (stainless-steel probe) & peel strength (stainless-steel)	Hai, et al. [104]
		10	Decrease (1310 ± 29 g)	Increase (1214 ± 79 12.5 mm of patch length)		
		15	Decrease (1140 ± 48 g)	Increase (1809 ± 63 12.5 mm of patch length)		
Patch (Gelva 737 acrylic/Silicone Adhesive 7-4503)	Lauryl alcohol	0	7 N/mm^2^	30 N/25 mm width of patch	Tack force (stainless-steel probe) & peel strength (stainless-steel)	Taghizadeh, Soroushnia and Mohamadnia [93]
		6	Increase (70 N/mm^2^)	Increase (65 N/25 mm width of patch)		
		12	Decrease (8 N/mm^2^)	Increase (13 N/25 mm width of patch)		
Patch (Gelva 737 acrylic/Silicone Adhesive 7-4503/ PVP 27/23)		0	1 N/mm^2^	65 N/25 mm width of patch		
		6	Increase (67 N/mm^2^)	Same (65 N/25 mm width of patch)		
		12	Decrease (7 N/25 mm width of patch)	Decrease (43 N/25 mm width of patch)		
Patch (styrene-isoprene-styrene block copolymer 3620)	Liquid paraffin	26	0.31 ± 0.09	0.53 ± 0.11	Tack force (stainless-steel probe) & peel strength (bakelite plate)	Wang, Liu, Tang and Han [90]
		32.6	Increase (0.79 ± 0.2)	Increase (0.77 ± 0.08)		
		39	Increase (2.76 ± 0.14)	Increase (1.59 ± 0.14)		
		45.5	Increase (3.31 ± 0.23)	Decrease (1.34 ± 0.12)		
Patch (deproteinised natural rubber latex)	-	Control	1.51 ± 0.51	0.10 ± 0.01 N/cm^2^	Tack force (stainless-steel) & peel strength (polyvinyl chloride surface)	Pichayakorn, et al. [105]
	Sodium CMC	15 parts per hundred rubber	Increase (1.86 ± 0.05)	Decrease (0.07 ± 0.01 N/cm^2^)		
	Sodium CMC & dibutylphthalate	15:10 parts per hundred rubber	Increase (1.79 ± 0.15)	Decrease (0.08 ± 0.01 N/cm^2^)		
Patch (styrene-isoprene-styrene block copolymer 3620)	Liquid paraffin	0.2	1.72 ± 0.14	2.81 ± 0.05	Tack force (stainless-steel probe) & peel strength (bakelite plate)	Wang, Ma, Liu, Han and Tang [17]
		0.3	Increase (3.21 ± 0.27)	Increase (4.71 ± 0.27)		
		0.35	Increase (1.79 ± 0.16)	Decrease (2.11 ± 0.17)		
		0.5	Decrease (0.93 ± 0.09)	Decrease (0.61 ± 0.07)		
Methoxyflavones (7% *w*/*w*) patch (DURO-TAK^®^ 2853)	-	Control	2.75 ± 0.07 kg	8.50 ± 0.05 N	Tack force (stainless-steel probe) & peel strength (stainless-steel)	Tuntiyasawasdikul, Limpongsa, Jaipakdee and Sripanidkulchai [92]
	IPM	3	Increase (3.06 ± 0.13 kg)	Decrease (8.32 ± 0.16 N)		
	Menthol and N-methyl-2-pyrrolidone	3:3	Increase (2.95 ± 0.19 kg)	Decrease (8.30 ± 0.37 N)		
	Menthol and PG		Increase (3.30 ± 0.03 kg)	Decrease (7.81 ± 0.10 N)		
	Oleic acid, menthol and N-methyl-2-pyrrolidone	3:3:3	Increase (2.99 ± 0.05 kg)	Decrease (6.82 ± 0.20 N)		

**Table 6:** Drug release kinetics models of MS-loaded patches with different solvents (PG: propylene glycol; TRC: Transcutol^®^ P; MAI: Maisine^®^ CC; IPM: isopropyl myristate; MS: methyl salicylate; MS-P: patches with drug but without solvent; -P: patches with drug and solvent).

Formulation	Zero Order Model	First Order Model	Higuchi Model	Korsmeyer-Peppas Model	
	*R^2^*	*R^2^*	*R^2^*	*R^2^*	*n*
MS-P	0.713	0.723	0.878	0.932	0.579
PG-P	0.732	0.755	0.889	0.931	0.615
TRC-P	0.858	0.891	0.954	0.959	0.680
MAI-P	0.855	0.854	0.955	0.966	0.716
IPM-P	0.783	0.809	0.923	0.947	0.647

## Data Availability

Not applicable.

## Supplementary Materials

The following supporting information can be downloaded at: https://www.mdpi.com/article/10.3390/pharmaceutics14112491/s1, Figure S1: Images of selected MS-loaded patches with and without solvents. (PG: propylene glycol; TRC: Transcutol® P; MAI: Maisine® CC; IPM: isopropyl myristate; PLU: Plurol® oleique CC 497; LA: Labrasol®; MS: methyl salicylate; MS-P: patches with drug but without solvent; -P: patches with drug and solvent); Figure S2: ATR-FTIR spectra of patches in the region of 1200–1000 cm−1; Table S1: Miscibility study of 10% *v*/*v* of MS in different solvents over 24 h.
