# Supplementary material for: Development and Characterisation of a Topical Methyl Salicylate Patch: Effect of Solvents on Adhesion and Skin Permeation
Source: Pharmaceutics. 2022 Nov 17;14(11):2491. doi: 10.3390/pharmaceutics14112491 (PMC9698037; doi:10.3390/pharmaceutics14112491)
Supplement: Supplementary file 1 [file pharmaceutics-14-02491-s001.zip › pharmaceutics-2017272-supplementary.pdf]

Supplementary information:

Development and characterisation of a topical methyl salicylate patch: Effect of solvents on adhesion and skin permeation

Soo Chin Yeoh [1], Poh Lee Loh [2], Vikneswaran Murugaiyah [3,4] and Choon Fu Goh [1,*]

[1] Discipline of Pharmaceutical Technology, School of Pharmaceutical Sciences, Universiti Sains Malaysia, Minden 11800, Penang, Malaysia; soochin.yeoh@student.usm.my

[2] THP Medical Sdn Bhd, 1209, Jalan Perindustrian Bukit Minyak 18, Kawasan Perindustrian Bukit Minyak, Simpang Ampat 14100, Penang, Malaysia; rd@teonghuat.com.my

[3] Discipline of Pharmacology, School of Pharmaceutical Sciences, Universiti Sains Malaysia, Minden 11800, Penang, Malaysia; vicky@usm.my

[4] Centre for Drug Research, Universiti Sains Malaysia, Minden 11800, Penang, Malaysia

* Correspondence: choonfugoh@usm.my

Correspondence:

Choon Fu Goh, Discipline of Pharmaceutical Technology, School of Pharmaceutical Sciences, Universiti Sains Malaysia, 11800 Minden, Penang, Malaysia. email: choonfugoh@usm.my

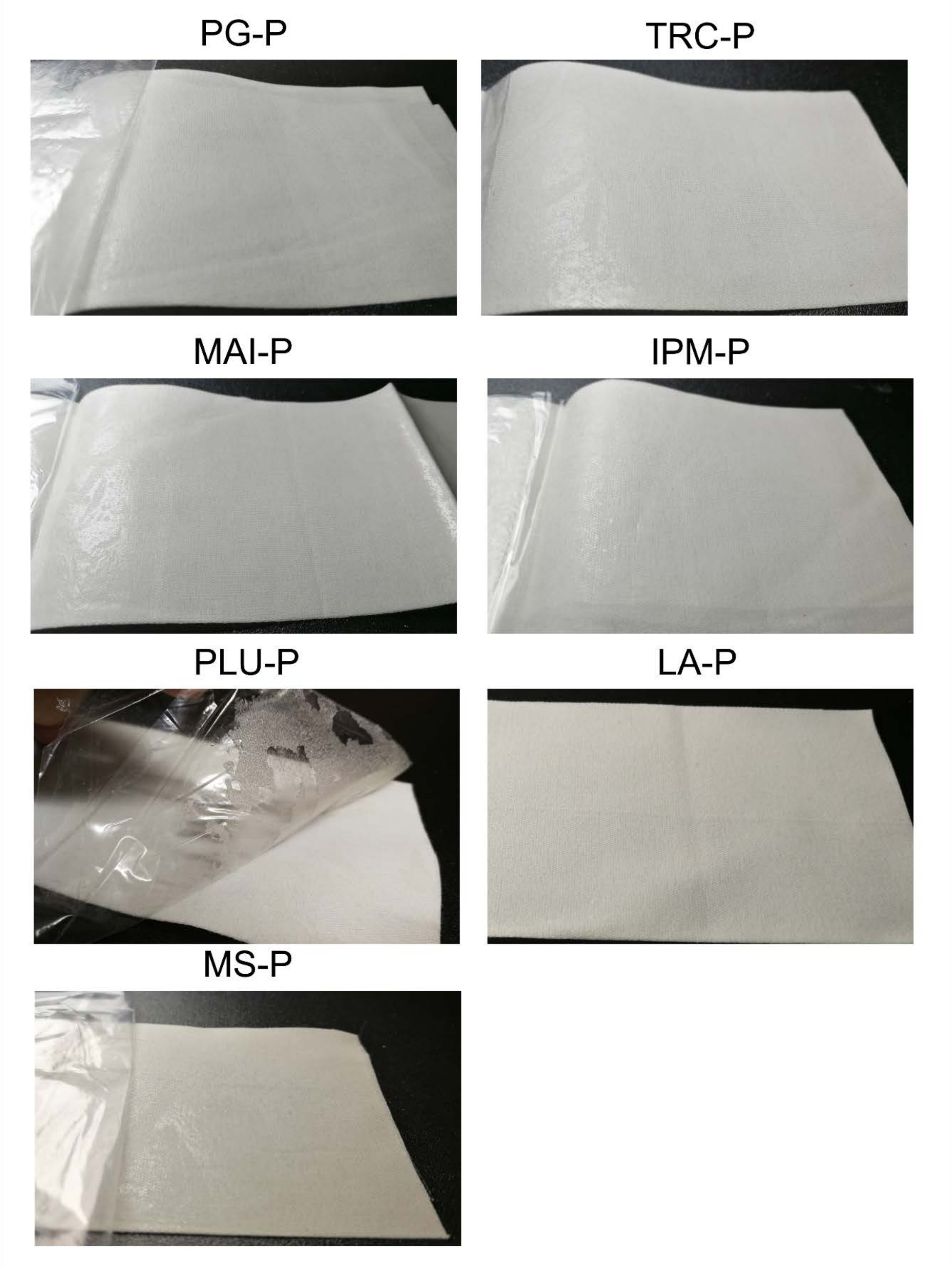

Figure S1. Images of selected MS-loaded patches with and without solvents. (PG: propylene glycol; TRC: Transcutol® P; MAI: Maisine® CC; IPM: isopropyl myristate; PLU: Plurol® oleique CC 497; LA: Labrasol®; MS: methyl salicylate; MS-P: patches with drug but without solvent; -P: patches with drug and solvent)

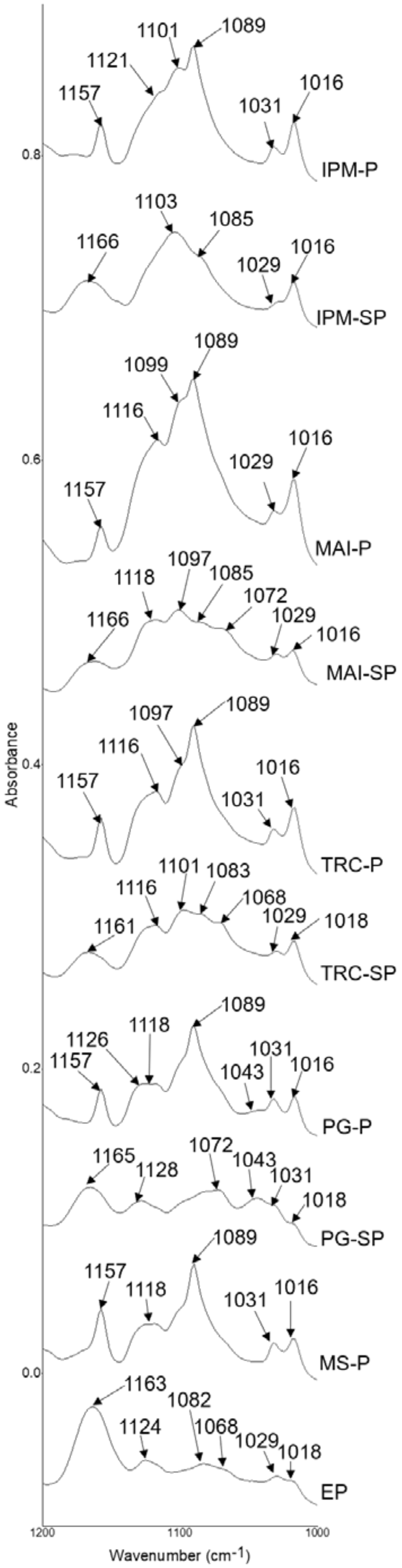

Figure S2. ATR-FTIR spectra of patches in the region of 1200 – 1000 cm^{-1}

Table S1. Miscibility study of 10%*v/v* of MS in different solvents over 24 h

Time (h)	Type of solvent					
	PG	IPM	TRC	LA	MAI	PLU
0	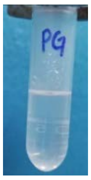	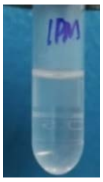	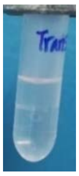	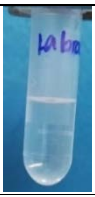	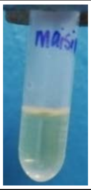	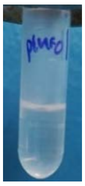
1	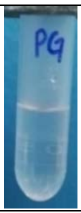	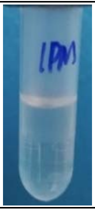	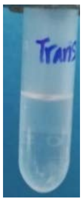	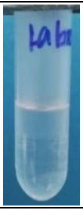	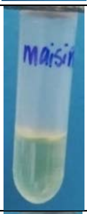	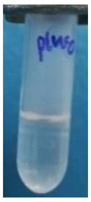
2	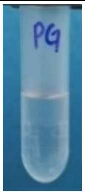	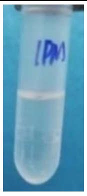	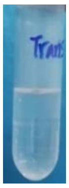	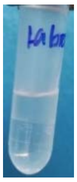	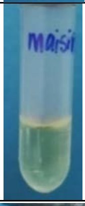	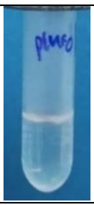
8	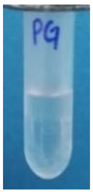	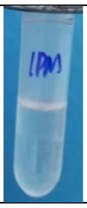	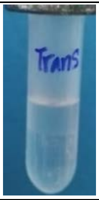	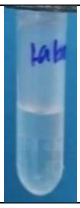	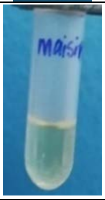	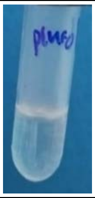
24	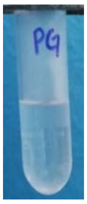	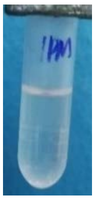	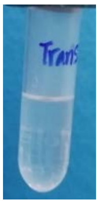	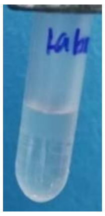	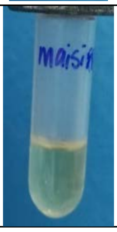	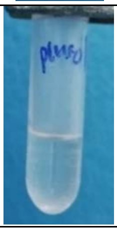
